# An 8-week physical exercise intervention for e'athletes improves physical performance rather than short-term esports performance parameters - a randomized controlled trial

**DOI:** 10.3389/fspor.2024.1504205

**Published:** 2025-01-21

**Authors:** Felix Wachholz, Nicole Gamper, Martin Schnitzer

**Affiliations:** Department of Sport Science, University of Innsbruck, Innsbruck, Austria

**Keywords:** physical activity, overall coordination, gaming, anticipation, reaction, skill, performance, grip strength

## Abstract

**Introduction:**

Esports have been suggested to enhance hand-eye coordination, fine motor skills, and reaction times. While physical exercise is known to improve these abilities, its impact on short-term esports performance is under-researched. This study aims to evaluate the effects of specific and general physical training on gaming performance, hypothesizing improvements in both physical and gaming-related variables.

**Methods:**

Baseline and follow-up tests measured global coordination, grip strength, Aimlabs performance, single and 4-fold reaction times, and anticipation. Participants completed a weekly and an ending questionnaire. Twenty-eight participants (12.3 ± 10.1 h gaming/week) were assigned to specific (*N* = 10), general (*N* = 9), and no training (*N* = 9) groups for an 8-week intervention. Variable changes over time and between groups were analyzed using a repeated measures ANCOVA.

**Results:**

Training significantly improved the overall coordination of those participants engaging in physical training compared to the control group [F(2,25) = 3.858, *p* = 0.035, *η*^2^ = 0.236]. Grip strength also showed significant improvement [F(2,25) = 6.084, *p* = 0.007, *η*^2^ = 0.327]. There were no significant time or group effects for the gaming variables, but the hours played by participants positively influenced Grid Shot [F(1,24) = 4.746, *p* = 0.039, *η*^2^ = 0.165] and Track Point [F(1,24) = 9.613, *p* = 0.005, *η*^2^ = 0.286] performance. The weekly questionnaire indicated a significant improvement in participants' well-being.

**Discussion:**

While training improved physical variables, no effects were observed in suggested short-term performance. However, gaming hours and experience during the intervention positively influenced performance. Future research should consider the amount of time spent playing as it may affect short-term performance. Furthermore, future research should strive to differentiate between various types of esports performance, such as short-term vs. long-term outcomes. Participants reported that engaging in physical activity within the context of esports and gaming positively contributed to their overall well-being.

**Clinical Trial Registration:**

ClinicalTrials.gov, identifier (NCT06264375).

## 1 Introduction

Esports refers to the competitive realm of playing digital games against a human opponent (1). Beyond this rational definition, it is a phenomenon which has garnered significant attention in recent years, driven by the exponential growth in the number of elite and highly ambitious casual e'athletes (2, 3). Despite the rise of esports and the similarity of the terms “sport” and “esports”, there is controversy over the status of esports in society and its connection to traditional sport (4–6), arguably due to the lack of visible movement while performing esports or video gaming. Video gaming is primarily a recreational activity that cultivates many of the skills essential for esports, but without the mainly competitive approach as seen in professional esports (7). Its influence is particularly significant among younger generations, shaping their behaviors, interests, and digital competencies (8). As in other sports, amateur or casual e'athletes might be influenced by elite e'athletes as role models (9). It is often argued that esports and video gaming can potentially cause acute and chronic health risks due to prolonged periods of sitting and excessively repetitive movements (10).

Research on the effects of gaming on physical abilities suggests that video gaming supports fine motor skills (11), hand-eye coordination (12) and enables e'athletes to perform up to 400 actions per minute using their keyboard and mouse (13, 14). Other studies highlight elite-level reaction times to visual information, as well as enhanced cognitive and strategic abilities maintained over hours (15, 16). In summary, performing gaming and esports over years appears to impact human abilities, suggesting that specific skills are necessary for superior performance, similar to traditional sports.

Research about the effect of general physical activity on gaming performance suggests that even a 6-minute rest, including a non-sitting activity between games, could improve processing speed and executive functions (10). This might be beneficial for e'athletes during their games and competitions and have short and long-term health benefits. Further positive short-term effects of physical activity on gaming performance have been shown recently, with e.g., short bouts of high-intensity interval training (17) or sprint exercises (8) improving esports performance. However, clear parameters defining esports performance have yet to be established, highlighting the need to identify valid and reliable variables for predicting performance in this field.

That said, research has employed various approaches to assess gaming or esports performance, including evaluating performance within specific game modes (10, 17) or actual competitive performance (18). Measurements to evaluate esports or gaming performance were mostly conducted over a prolonged time period, measuring performance over many minutes or even hours. An exception is the study by Manci et al. (2024), which evaluated the effects of acute exercise on short-term esports performance. The performance metric involved the time required for participants to destroy 50 randomly appearing and moving opponent targets, with completion times ranging between 110 and 154 s. They found significant effects of acute physical exercise on gaming performance and cognitive abilities, arguably due to various neurobiological mechanisms. Another study evaluated that an 8-week intervention might help to reduce fatigue, both mental and physiological, which again highlights the potential benefit of physical activity for esports and gaming (19). Another example is a 10-week treadmill-based exercise intervention, which significantly improved cardiovascular fitness, reaction times, cognitive accuracy, and heart rate variability in elite e'athletes, while also influencing cerebral oxygenation levels, suggesting that structured aerobic training can enhance physiological and cognitive performance in this population (20). However, a recent systematic review by McNulty et al. highlighted the possible positive effects of physical activity on esports performance, but also stated that “more controlled experimental evidence is needed to investigate short- and long-term effects of exercise on in-game performance” (21) (p. 9). There is a notable gap in research regarding the specific physical and trainable motor skills required in esports, as well as the potential benefits of targeted training programs designed to enhance these abilities for e'athletes (3, 22). It is essential and highly intriguing to explore potential synergy effects of this nature, as evidence-based knowledge on the subject remains limited. Furthermore, with the growing number of e'athletes, it becomes increasingly important to implement measures that prevent injuries, enhance performance, and promote overall health within this relatively young and rapidly expanding community (23, 24).

One example is the effect of gaming on cardiovascular and respiratory parameters like heart rate, respiratory rate and minute ventilation, which showed an increase during acute gaming (25, 26), as well as perceived exhaustion (27). Kocak (2022) showed that energy expenditure during gaming is in fact 40% higher than during sitting and that physical fitness does indeed have an impact on performance. However, it is still unclear whether a generally higher level of physical fitness has a measurable effect on short-term gaming performance.

Another example of potential synergies regarding physicality in esports is hand-grip strength, as evidence suggests that higher grip strength improves dexterity in elderly people (28). A possible mechanism is improved intramuscular coordination, which could be indicated by the higher force values and therefore be responsible for a better neuromuscular harmonization within the muscle (29). On the other hand, video gaming does improve manual skills and grip strength of the non-dominant hand (30). Hence, enhanced dexterity could significantly improve the ability to execute game-specific actions more quickly and accurately, particularly in genres that demand precise and rapid finger movements (13, 31). Other, especially coordinative abilities such as reaction time are skills that are often named first in the context of esports and gaming and have been shown to be similar for traditional sport athletes and e'athletes (32). While the trainability of reaction times remains a topic of debate, evidence suggests that both traditional sports and esports contribute to faster reaction times. Beyond reaction times, anticipation is also considered a critical factor in gaming performance, with studies indicating that individuals with video gaming experience demonstrate superior anticipation skills and greater consistency compared to peers without such experience (33). However, the possible effect of training that specifically focuses on reaction times or anticipation and its effect on esports and gaming performance still needs to be evaluated.

Based on current knowledge, the aim of the proposed study is to evaluate the effect of a specific and a general physical activity training intervention on (i) physical parameters like overall cardiovascular fitness and grip strength, as well as (ii) short-term gaming performance, reaction time, and anticipation, which have been suggested to be important for good performance in esports and gaming. Therefore, it was hypothesized that a physical training regimen that promotes cardiovascular performance and various neurobiological mechanisms might lead to better gaming performance as suggested by recent literature (8, 21). Moreover, one assumption was that increasing grip strength might improve the motor control of hand movements (28). Since e'athletes tend to overestimate their physical activity level (34), the subjective effect of a physical training regimen on performance and well-being was investigated in addition to these objectively measurable parameters.

## 2 Methods

### 2.1 Participants

Recruitment and first measurements for the study started in January 2024 and was conducted using the university newsletter and personal contacts to esports clubs. The last follow-up measurements were conducted in April 2024. Since we were interested in semi-professional and casual e'athletes, inclusion criteria were that participants played at least 5 h per week on a regular basis (8, 15), with the overall average being 12.3 ± 10.1 h of playing per week. Exclusion criteria included diagnosed visual impairments, depression, neurological, psychiatric, orthopedic or cardiovascular disease or taking medication before or during the study period (8). The overall age of the sample consisting of 28 participants was 27.5 ± 8.3 years and their BMI was 23.2 ± 2.8 kg/m^2^. The three different intervention groups, Specific Training Group (*STG*, *N* = 10), General Training Group (*GTG*, *N* = 9) and No Training Group (*NTG*, *N* = 9) did not differ in terms of their age, BMI or the number of hours they played their games weekly (Table 1). All participants were male, primarily right-handed (*N* = 2 left-handed), with 57.1% having right eye dominance. To determine the required sample size, the G-Power 3.1.9.7 software (Heinrich Heine University Düsseldorf, Germany) was used a-priori (ANOVA: repeated measures, within-between interaction). To the best of our knowledge, there has not been a study that describes results related to physical exercise and gaming or esports variables using a repeated measures ANCOVA. Hence, based on a prior study reporting a Cohen's d of approximately 0.5 for the effects of physical exercise on cognitive performance in e'athletes (17), we used a similar approach to Manci et al. (2024), with an effect size *f* = 0.25, an alpha (error) rate of 5% and power of 80%, but three instead of two groups, to estimate the required sample size for a repeated measures ANOVA with four different measures in the three groups. The determination in G-Power yielded a total sample size of 30 participants, which we reached when starting the measurements. However, due to two drop-outs we ultimately analyzed 28 participants. Most of the participants played First-Person-Shooter (FPS)-games (*N* = 18), followed by simulation games (*N* = 7) and multiplayer online battle arena (MOBA) games like League of Legends (*N* = 3). All games included require precise clicks, fast reaction times, and good anticipation in order to perform well. Participants were instructed to abstain from nicotine on the day of their measurements, and to avoid heavy training or alcohol consumption the day before.

**Table 1:** Comparison of the participants' demographic data within the different groups.

	*STG* [*n* = 10]	*GTG* [*n* = 9]	*NTG* [*n* = 9]	*p*-value
Age [years]	28.7 ± 8.2	27.4 ± 10.0	26.3 ± 7.4	0.836
BMI [kg/m^2^]	23.6 ± 3.0	23.2 ± 3.0	22.7 ± 2.6	0.822
H/week [h]	13.9 ± 7.6	14.1 ± 10.6	8.7 ± 12.1	0.448
Started - no. of years ago	16.3 ± 9.1	16.3 ± 7.0	14.8 ± 7.1	0.889

STG, Specific Training Group; GTG, General Training Group; NTG, No Training Group.

All participants were informed about the measurement procedures, about any possible risks involved and were asked to provide written informed consent regarding their participation. Participants were free to withdraw from the experiment at any time without reason. Prior to any measurements, the study had been approved by the Board for Ethical Questions in Science at the University of Innsbruck (Certificate 03/2024). All measurements and the use of equipment and procedures were performed in accordance with the Declaration of Helsinki (1964).

### 2.2 Study design

**Figure 1:**
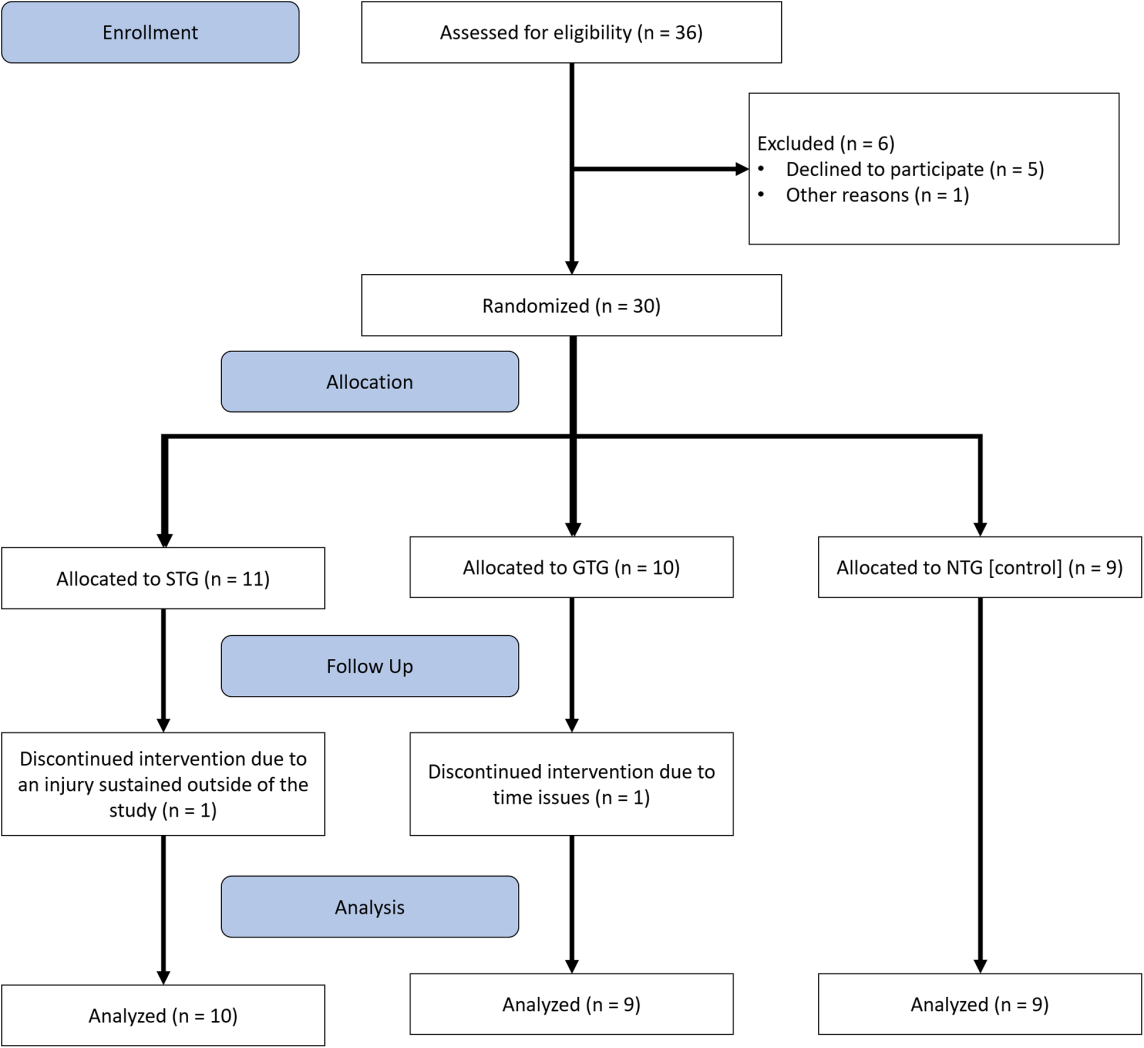
Visualization of the used study design following the CONSORT guidelines.

Using a parallel randomized controlled trial approach, visualized in Figure 1, participants were assigned to one of the groups (*STG*, *GTG* and *NTG*) using stratified randomization to ensure that the pre-study weekly hours played did not differ between the groups so as to maintain comparability between the three groups (35). The protocol of this intervention study was pre-registered on ClinicalTrials.gov (ID NCT06264375) and neither assessors nor participants were blinded regarding the group allocation. Pre- and post-tests were performed for every participant at the same time of day after approximately 8 weeks of intervention (on average 56.9 ± 2.2 days between pre- and post-test). The two interventions differed in terms of the kind of training and focus received, but the training load and intensity were similar. On average, they trained three times per week for approximately 40 min each session (for a detailed overview of the training regimen, please refer to “Data Sheet 1”, which pertains to the General Training Group (GTG), and “Data Sheet 2”, which provides information on the Specific Training Group (STG), both available in the Supplementary Material). To track the training workload, a weekly questionnaire was used and all the information regarding the training was handed to them on a USB stick, containing the intervention plans, videos and precise exercise descriptions. Moreover, the smartphone application CoachNow (36) was used to stay in contact with the participants during the intervention period. No feedback on performance was provided. The mentioned videos were also available on the app.

#### 2.2.1 Specific training group [STG]

For the *STG*, the training focused on high intensity interval training (HIIT) for time-efficient sessions that met the group's needs (37, 38). HIIT is ideal for heterogeneous groups as it allows individuals of varying fitness levels to work at their maximum capacity, ensuring both fit and less fit participants achieve the intensity needed for performance improvements within the same standardized session (39). To ensure ease of use and to maintain a low barrier to participation, participants were instructed to use the Tabata Timer app (40), accompanied by clear guidelines on how to configure the intervals for their training. This approach minimized complexity and logistical effort, supporting consistent adherence to the program. The training consisted of whole-body movement, interrupted by a short break which did not, however, allow the participants to fully rest, followed by another whole-body exercise and a break. These patterns remained similar throughout the training sessions, following the concept of HIIT (41). To add training of several esports-specific abilities, the participants' reactions were trained on the participants' smartphone using the “LightsOut Reaction Time” app (42). The training using these apps was added to the physical training, that is, performing, e.g., lunges, while playing the mentioned app on their smartphones. Similarly, a Stroop test was implemented using the “Color Challenge” app (43), which the participants played during their physical exercise. Additionally, exercises with falling balls and objects with a trajectory were implemented to train anticipation. Training also included videos showing a dot traveling from one side to the other, with participants clapping their hands when the dot crossed a visible line, all while performing exercises such as lunges. Grip strength was trained following a typical strength training regimen (44), containing four bouts of intensity, e.g., squeezing a ball as hard as possible 12 times, holding the squeeze for 3–4 s and 2 min of rest between the sets.

#### 2.2.2 General training group [GTG]

For the *GTG*, training also focused on HIIT. Like the *STG*, participants were instructed to use the Tabata Timer app (Tabata Timer, V.33.1.4, 2024) and given specific interval settings for their workouts. The training involved the same full-body exercises with short breaks that prevented complete rest, followed by another set of full-body exercises and another short break. Again, this pattern remained consistent throughout the sessions, adhering to the HIIT concept (Machado et al., 2019). The *GTG* incorporated one more round of HIIT, so as to make it differ from the *STG* intervention as no other training was implemented in their regimen. The intervention plans (“Data Sheet 1”, containing details about the General Training Group (GTG), and “Data Sheet 2”, outlining information for the Specific Training Group (STG)) are available in the Supplementary Material.

#### 2.2.3 No training group [NTG] – control

**Figure 2:**
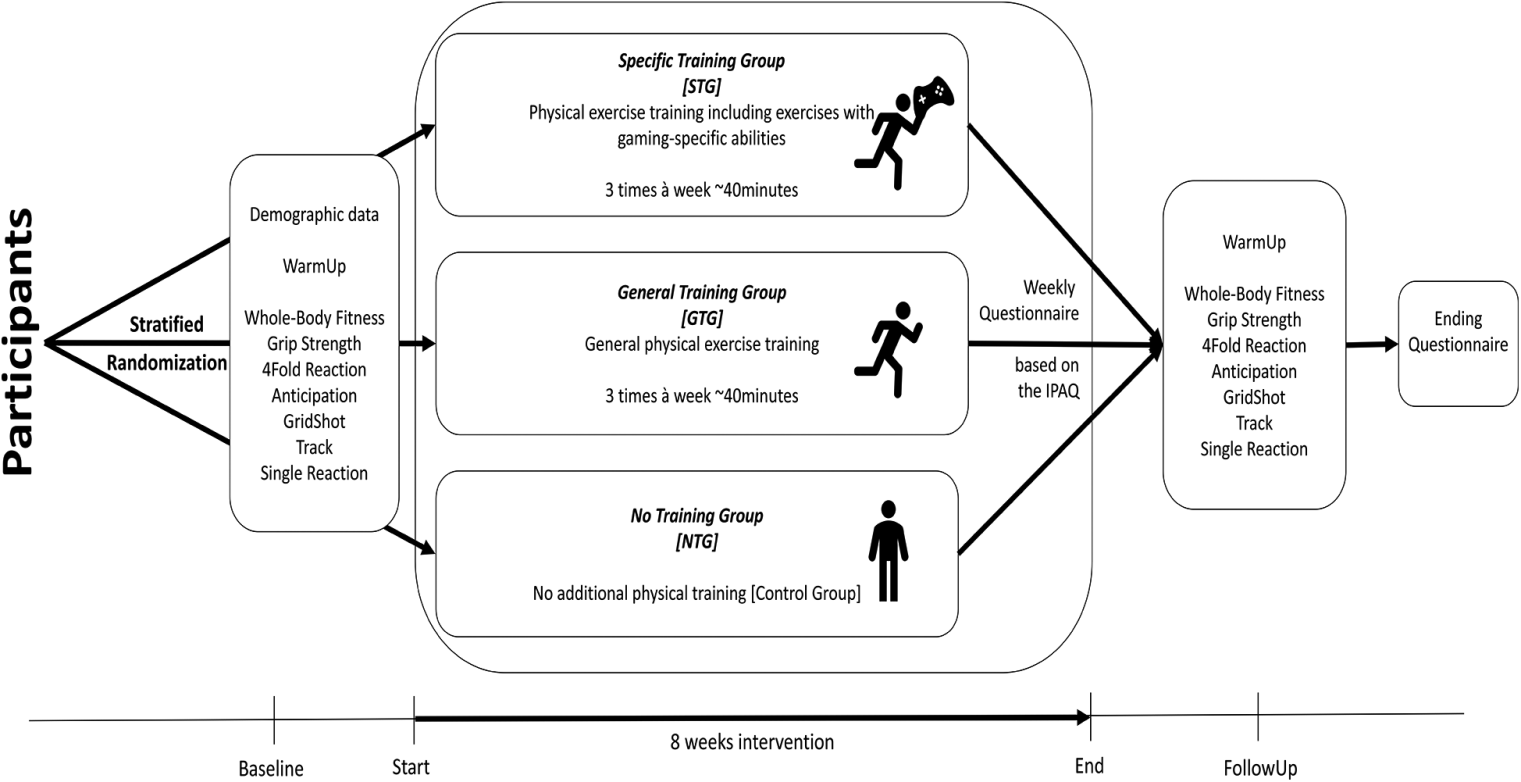
Visualization of the study design presenting the 8-week course of intervention.

The *NTG* did not train in any specific way but was asked to maintain typical behavior and answer the weekly questionnaire. The study procedure is visualized in Figure 2.

### 2.3 Variables

Participants warmed up before taking the standardized baseline and follow-up measurements so as to be ready for the following assessments which were always conducted in the same order. The warm-up contained three minutes of easy running *in situ*, ten squats, ten lunges, finger rows while doing skate jumps, squeezing of the fingers and five explosive squat jumps to prepare them for the high intensity of the overall coordination and grip-strength assessment. Besides warming up, the procedure allowed for the participants' movement to be corrected where necessary. For example, if squats were not performed correctly, participants received feedback. This was important since the warm-up exercises were also part of the training regimen.

#### 2.3.1 Physical variables

Participants underwent a test similar to a four-corner shuttle run (45, 46) as an all-round coordination test focusing on agility, endurance and explosive strength using BlazePods (47). Four BlazePods formed a square, each placed at a corner with a 1.5-m gap. Participants started between two BlazePods, which randomly lit up in blue. The participants aimed to strike as many BlazePods as possible in 60 s, using only their right hand for the two on their right and their left hand for the two on their left, without turning around. The number of BlazePods struck was used for analysis.

Since most participants were right-handed and all used the computer mouse with their right hand, their right-hand grip strength was measured using the Grip Strength Dynamometer 5401 (Takei, Tokyo, Japan). Participants sat with their forearms on a table and elbows just behind the table's edge, thumbs facing up. They squeezed their hand as hard as possible, moving the thumb toward the fingers. The maximal score from two trials was recorded, following the Southampton protocol (48).

#### 2.3.2 Gaming variables

To evaluate the participants' esports performance, the Aimlabs software (49) was used (50). During the tests, participants maintained an upright sitting position with their left hand positioned on the keyboard and their right hand operating the mouse (Corsair Gaming, Inc., USA), as deemed crucial for test accuracy. The mouse's dots per inch (dpi) were consistently set to 1,500 dpi to ensure uniformity of results and the mouse was used on a mousepad. Additionally, participants used the same mouse throughout the study to facilitate comparability (51). The distance to the 26.5″ and 2,560 × 1,440 pixel resolution screen (ASUSTeK, Taiwan, OLED monitor and 0.03 milliseconds response time) was maintained at approximately 70 cm (8, 52), while the screen's framerate was always set to 240 Hz. During the Aimlabs and reaction tests, participants wore a headset to block noises from their surroundings.

The first Aimlabs test was Grid Shot, where participants clicked three balls appearing randomly in a virtual 3D environment. The light blue targets, sized 100 pixels, appeared randomly, and participants had to click on as many as possible in 60 s. Performance was measured in points, accounting for the number of targets and accuracy [F(3,27) = 4,747.64, *p* < 0.001; target (*t* = 26.636, *p* < 0.001); accuracy (*t* = 17.305, *p* < 0.001); corrected R^2^ = 0.998, f^2^ = 499]. Reaction time was assessed but did not significantly influence points (*t* = −0.764, *p* = 0.453). Each participant had two trials with a two-minute rest between them. Figure 2 shows a snapshot of the test.

In the second Aimlabs test, an orb repeatedly strafed from left to right at random, testing the participants' ability to track movement and keep the crosshair on the target. The orb's size adaptively increased or decreased based on how well the participants were performing. The resulting performance points used for analysis were generated by the relation of the crosshair being on or off the target [F(3,27) = 1,090.19, *p* < 0.001; (*t* = 13.157, *p* < 0.001); Corrected R^2^ = 0.992, f^2^ = 124]. Again, each participant had two attempts of 60 s, with a two-minute rest period between them. A picture of the measure is shown in Figure 2.

To test isolated reaction times, a 4-fold reaction test was used. Participants faced a black panel with red diodes at each corner and buttons under each hand and foot. The diodes lit up randomly, and participants had to press the corresponding button as quickly as possible. For the upper corners, they used their hands (left for left, right for right) and for the lower corners, they used their feet (left for left, right for right). After one familiarization round, each round consisted of ten lights, and the average reaction time from three rounds was recorded and calculated using LabView software (53). Figure 2 shows the procedure.

**Figure 3:**
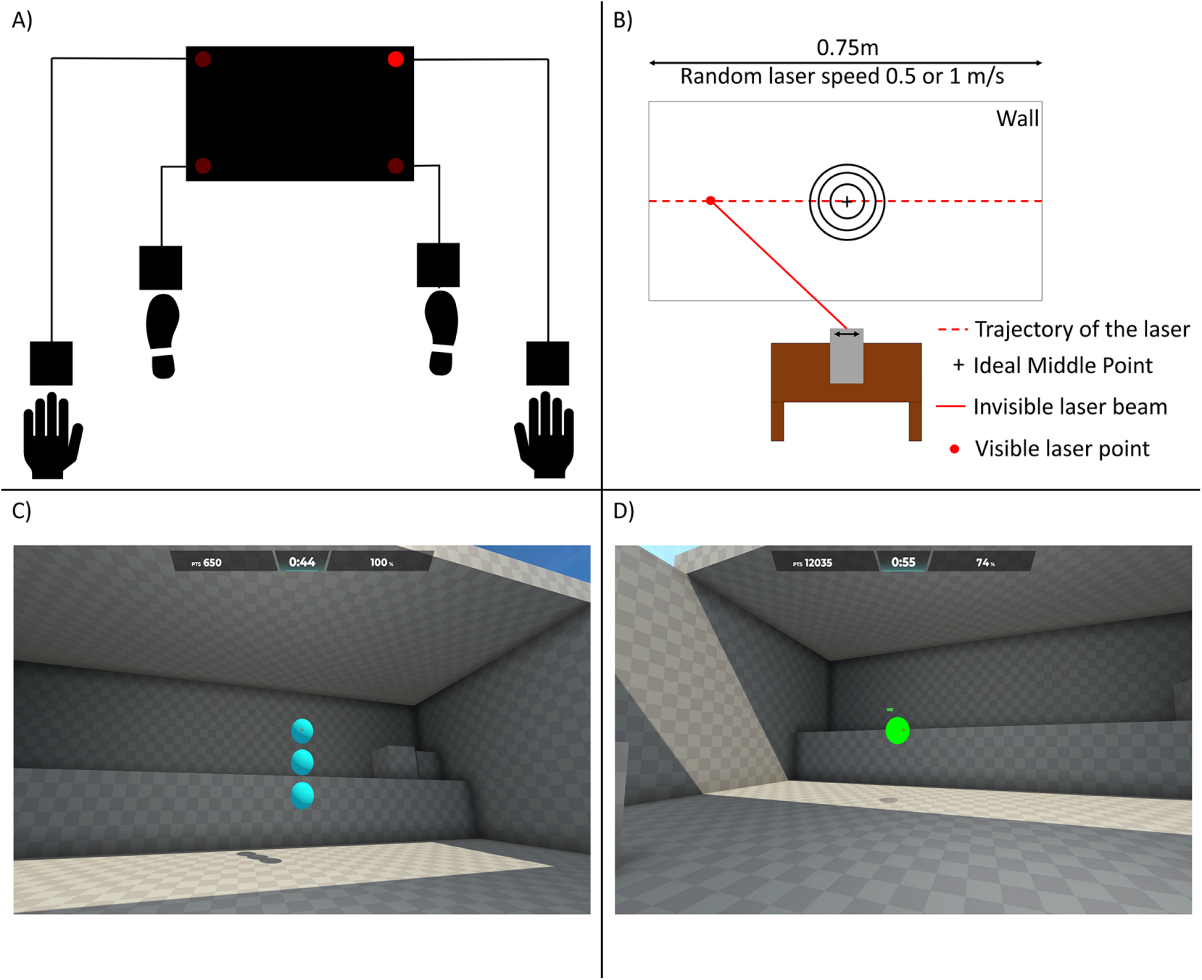
Measurements of the gaming performance variables. **(A)** shows a diagram of the 4fold reaction test. A diode is positioned in each corner of the panel which lights up randomly. In the diagram, the right upper corner is lit up, indicating that the participant would have to press it with the right hand. **(B)** Participants had to click a button just as the laser reached the ideal (middle) point of the traveling dot. **(C)** Screenshot of the Grid Shot task in Aimlab. The aim was to click away the balls as soon as they appeared. The point tally, remaining time and accuracy are displayed at the top. **(D)** The same environment as in C was used for the Track Point task, in which participants had to follow the orb wandering from left to right by placing the crosshair on the target, which moved randomly.

To test anticipation, a laser-based test was used with a laser moving horizontally at random speeds (0.5 m/s and 1 m/s) (54, 55). After three practice runs, participants had to press a button when the laser reached the midpoint marked on the wall. The laser traveled 75 cm, and participants sat 80 cm away. A LabView (53) software recorded the timing differences for ten trials, noting clicks that were too early or too late. Dependent variables were the number of accurate hits and the average timing deviation from the midpoint in three rounds. A scheme of the test is presented in Figure 3.

Lastly, single reaction time was tested using the web browser application AimBooster (56). A custom code was used, setting the target size to 430 pixels on full screen. The target appeared at random times and needed to be cleared by clicking on it. Participants did not need to move their mouse due to the large size of the target. They were seated upright at a consistent distance from the screen. The reaction time taken to click away the target was measured over a 60-second period, recorded, and the mean reaction time was calculated.

### 2.4 Weekly questionnaire

To keep track of the participants' activities during the intervention period, they were asked about their physical activity level on a weekly basis using a German version of the International Physical Activity Questionnaire (IPAQ) (57). The questions aimed to determine the number of days participants engaged in intense physical activity each week and the duration in hours, their moderate physical activity habits, the days and duration of their walking activities, and the amount of time they spent sitting, likewise expressed in hours. Moreover, they were asked how many hours they had spent playing virtual games in the past week and how they felt before, during and after their training session in terms of their well-being using a modified 7-point Borg scale (58) featuring happy or sad smileys. In case participants forgot to fill out the questionnaire, they were reminded by the authors using the CoachNow app.

### 2.5 Ending questionnaire

Besides asking the participants about their weekly behavior, they were asked to fill out a final questionnaire on their smartphone at the end of the intervention after the last measurement. Participants were asked what kind of sport they did besides the training regimen, whether they would continue with the training, and whether they would like physical activity to be offered as part of the training at esports clubs. Moreover, participants were asked to reflect on how the training had impacted them subjectively. In total, five items were asked, focusing on how they felt their aim had been impacted by the training. Questions were presented in a randomized order and the mean for each item was calculated.

### 2.6 Statistical analysis

To test for differences between the groups regarding the physical activity and gaming performance variables, a repeated measure analysis of variance (rmANOVA) was used. Data were tested for normal distribution using a Shapiro-Wilk test and a Greenhouse–Geissler adjustment was performed to correct for violations of sphericity, which was tested using Mauchly's-test for sphericity. Post-hoc results were corrected using Bonferroni-correction. Moreover and in regard to the gaming performance variables, a repeated measure analysis of variance including the hours participants played during the intervention as a covariate was performed (rmANCOVA), as a higher number of played hours is considered to increase performance compared to fewer hours played (59). Although groups were matched in terms of hours played per week, actual hours played per week over an eight-week intervention period can vary. Additionally, even within a group, some participants play more, some less. The hours participants played per week were assessed using the weekly questionnaire. Homogeneity of regression slopes was not violated with regard to the dependent variable, as the interaction terms were not statistically significant (*p* > 0.05). Normal distribution of data was tested, again using Shapiro-Wilk.

The reported well-being on the 7-point Borg-scale before and after training was averaged over the 8-week intervention and the two groups *SGT* and *GTG* compared using a *t*-test for independent variables, after ensuring normal distribution of the data. And lastly, the five scales (aim, reaction, focus, rage and stress) of the ending questionnaire were tested for normal distribution. If valid, a *t*-test for independent variables was used to test for differences between *STG* and *GTG*, whereas a Mann-Whitney-U test was performed if normal distribution was violated. All statistical analyses were performed using SPSS (IBM SPSS Statistics, Version 27, SPSS Inc., Chicago, IL, USA) and the α-level for significance was set at 0.05. The effect sizes were reported as partial *η*^2^ for rmANOVA and rmANCOVA analyses.

## 3 Results

### 3.1 Variables

#### 3.1.1 Physical variables

**Figure 4:**
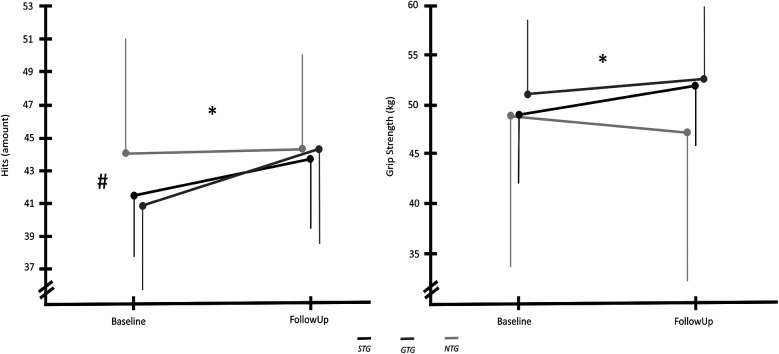
Number of hits the participants were able to accomplish (left) and participants’ produced grip strength (right) in the baseline and follow-up measurement. Vertical thin lines indicate the standard deviations. The asterisks (*) display a significant change between the groups over time. The hashtag (#) indicates significant differences over time.

Results regarding the overall coordination presented a significant alteration in the amount of BlazePods participants could tap out in 60 s [F(1,25) = 21.674, *p* < 0.001, *η*^2^ = 0.464]. In the two training groups, *STG* and *GTG*, the increase revealed significantly greater changes compared to the *NTG* [F(2,25) = 3.858, *p* = 0.035, *η*^2^ = 0.236], presented in Figure 4. Similar to overall coordination, grip strength showed higher force changes in the two training groups compared to the *NTG*, which displayed a decrease in force produced by squeezing their hand, [F(2,25) = 6.084, *p* = 0.007, *η*^2^ = 0.327], as visualized in Figure 4. However, no significant change in grip strength between baseline and follow-up [F(1,25) = 2.485, *p* = 0.127, *η*^2^ = 0.090] could be observed.

#### 3.1.2 Gaming performance variables

**Figure 5:**
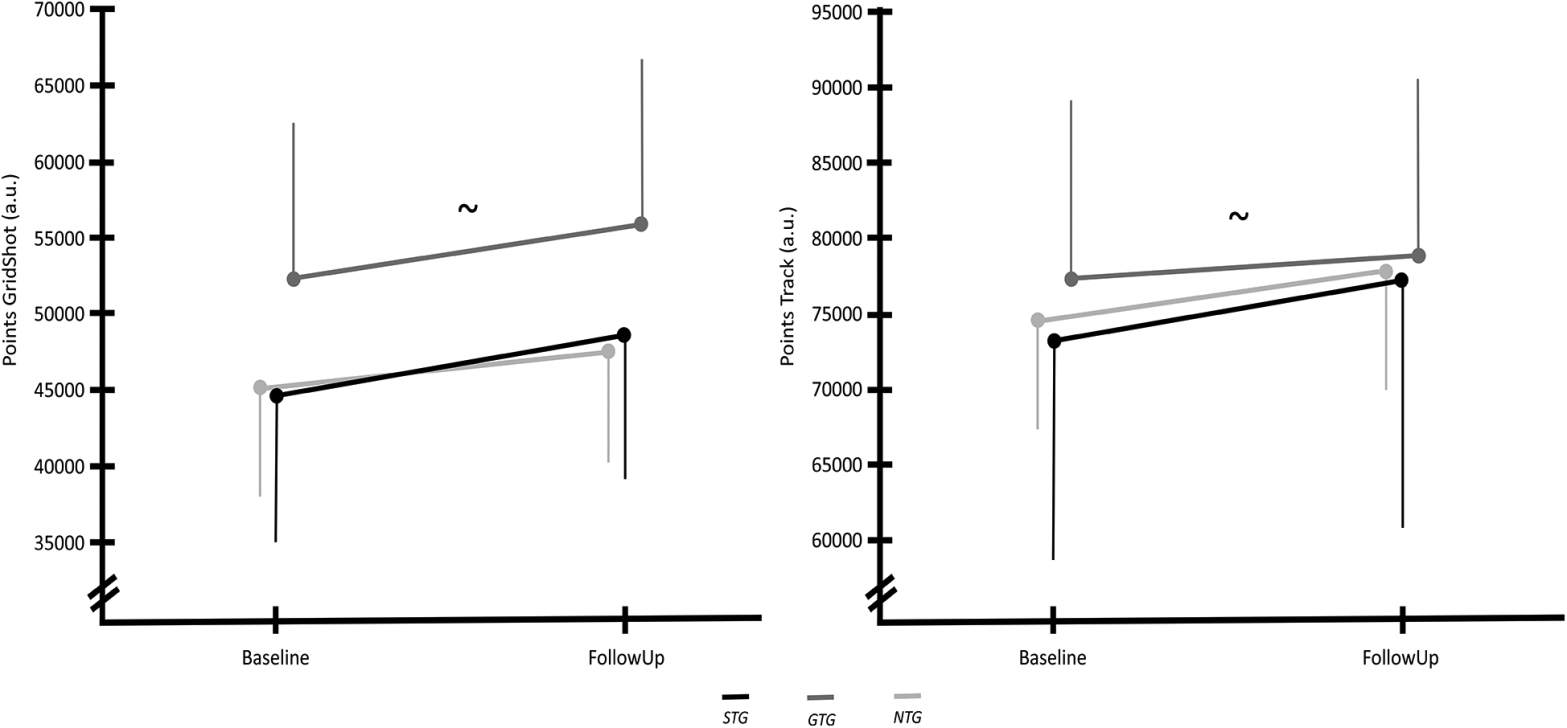
Number of points the participants were able to accomplish in the grid shot task (left) and the track point task (right) in the baseline and follow-up measurement. Vertical thin lines indicate the standard deviations. The tilde (∼) represents a significant effect of the number of hours played by the participants on the results.

The performance points of the Grid Shot test revealed a significant improvement between baseline and follow-up measurement [F(1,25) = 11.334, *p* = 0.002, *η*^2^ = 0.312], but no differences between the groups [F(2,25) = 0.198, *p* = 0.821, *η*^2^ = 0.016]. However, when using the played hours as a covariate, a non-significant increase in performance was observed in every group [F(1,24) = 0.133, *p* = 0.719, *η*^2^ = 0.005]. Moreover, no difference between the groups could be observed [F(2,24) = 0.371, *p* = 0.694, *η*^2^ = 0.030]. When tested for the number of hours played by the participants during the intervention, the results indicate that the hours played had a significant influence on the variable [F(1,24) = 4.746, *p* = 0.039, *η*^2^ = 0.165]. The results of the rmANCOVA can be seen in Figure 5.

Similarly, the performance-points of the Track Point measure exposed significant performance improvements in every group [F(1,25) = 6.058, *p* = 0.021, *η*^2^ = 0.195], but not between groups [F(2,25) = 0.356, *p* = 0.704, *η*^2^ = 0.028]. Again when checking for the number of played hours, the increase was no longer significant [F(1,24) = 0.683, *p* = 0.417, *η*^2^ = 0.028] and no difference between the groups could be detected [F(2,24) = 0.645, *p* = 0.534, *η*^2^ = 0.051]. When accounting for the participants' played hours during the intervention, the results indicated a significant influence of these hours on the variable [F(1,24) = 9.613, *p* = 0.005, *η*^2^ = 0.286]. The findings of the rmANCOVA are visualized in Figure 5.

None of the other suggested variables related to gaming performance presented significant changes with respect to the intervention [4Fold reaction F(1,24) = 0.919, *p* = 0.347, *η*^2^ = 0.037]; anticipation perfect hits [F(1,24) = 0.873, *p* = 0.359, *η*^2^ = 0.035]; anticipation average time [F(1,24) = 0.053, *p* = 0.820, *η*^2^ = 0.002]; and single reaction [F(1,24) = 0.004, *p* = 0.949, *η*^2^ = 0.000]. Moreover, no differences could be found regarding group differences or the effect of hours played.

### 3.2 Weekly questionnaire

Based on the subjective feedback from the Borg-scale before and after training, the *STG* reported that they felt significantly better after the training (5.7 ± 0.8), compared to their well-being before the training {4.7 ± 1.2 [t(9) = −2.507, *p* = 0.033]}. Similar results could be observed for the *GTG*, who reported a significant increase in well-being [t(8) = −2.239, *p* = 0.056] from before the training (4.2 ± 0.8) compared to after the training (5.1 ± 1.1). However, no significant difference could be detected between the groups [F(1,17) = 0.023, *p* = 0.880, *η*^2^ = 0.001]. Based on the IPAQ results, neither gaming behavior, nor physical activity behavior besides the intervention regimen changed notably during the 8-week intervention.

### 3.3 Ending questionnaire

The five items asked in order to determine how participants felt subjectively presented good internal consistency and included (i) aim (five questions, Cronbach's alpha = 0.884), (ii) reaction (five questions, Cronbach's alpha = 0.859), (iii) focus (three questions, Cronbach's alpha = 0.698), (iv) potential rage (four questions, Cronbach's alpha = 0.803) and (v) stress (five questions, Cronbach's alpha = 0.831). Results from the questionnaire showed no significant differences between *SGT* and *GTG* for the scales aim [t(16) = −1.541, *p* = 0.143], reaction [t(16) = −1.223, *p* = 0.239], focus [t(16) = 0.438, *p* = 0.668], rage [t(16) = 0.354, *p* = 0.728] and stress (*U* = 36.000, *p* = 0.730). When asked if participants would continue with the physical training ten said yes (3) or rather yes (7). Most of the participants who said rather no (6) said they would continue with other kinds of physical activity such as running or swimming. Only three participants said that they would not continue with the physical training. Regarding the question of whether they would like esports clubs to offer physical activity programs, 16 participants responded with “yes” and 11 with “rather yes”. None of the participants did not want physical activities to be part of esports club programs.

## 4 Discussion

The goal of the study was to evaluate the effect of specific and general physical training on participants' gaming performance. Although the specific and general physical training appeared to improve physical parameters, no effects could be observed in terms of the short-term esports performance parameters. More specifically, no differences between the groups could be observed in the Aimlabs performance tests, reaction times or anticipation. However, when adjusted for the hours participants spent playing during the intervention period, the effects became significant. It is noteworthy that the time spent gaming did not change during the 8-week intervention. Still, the time spent gaming and practicing gaming or esports in our sample appeared to have a significant effect on performance, which is in line with existing literature (33, 55). Hence, video game experience is associated with higher accuracy and consistency of anticipation, which might lead to an increase in performance. Even when drawing comparisons between the *STG* and *GTG*, parameters such as reaction times or anticipation remained unaffected. This finding should be considered in future research, as the amount of time spent playing might have a significant effect on short-term esports and gaming performance. Arguably, results could differ if the sample consisted solely of elite e'athletes, as improvement margins in this group might be smaller and remain unaffected by hours of play compared to casual e'athletes. After all, elite e'athletes already spend most of their day playing, especially esports games (60). Therefore, we propose that the number of hours played should always be checked when conducting intervention studies on esports and gaming performance. Moreover, the game genre played by participants in such an intervention study should be as consistent as possible, as the genre itself may influence performance parameters (61). In our study, the majority of participants played FPS games, which have been associated with superior sustained attention, reaction time, and inhibition abilities compared to, for example, MOBA players. However, our results indicate that effective physical training methods for esports still need to be developed, as no transfer effects from training focusing on reaction and anticipation to these esports performance related parameters were observed.

The lack of a significant effect of the physical activity intervention on short-term performance somewhat contradicts existing research (8, 10, 17, 19) and emphasizes the need for further research examining the impact of such an intervention on long-term esports performance, with evaluations spanning several minutes to even hours. The performance test used might have been too short, as cognitive and strategic abilities in esports often need to be maintained over hours (15, 16). The current study aimed to evaluate trainable physiological effects related to motor control and accuracy. Besides one recently published article (20), existing research has mostly focused on acute effects, but not the effect of a training regimen over several weeks. Building on the findings of Nicholson et al. (2024), a 10-week aerobic exercise intervention demonstrated significant improvements in participants’ cardiovascular fitness, as evidenced by increased time to exhaustion and heart rate deflection point. These physiological enhancements correlated with improved cerebral oxygenation and heart rate variability—key indicators of enhanced autonomic and neurophysiological functioning. While overall reaction times did not show significant improvement, specific gains were observed in incongruent reaction time tasks, highlighting subtle cognitive benefits. This discrepancy may reflect the nuanced relationship between aerobic fitness and the highly specialized cognitive skills required for esports performance, which may not be directly influenced by general physical fitness alone. Instead, the intervention likely bolstered broader cognitive functions such as sustained attention and inhibitory control, which contribute to long-term mental resilience and well-being rather than immediate, esports-specific performance metrics. Additionally, Nicholson et al. (2024) focused on professional e-athletes, a distinction that may explain differences in findings. Elite e-athletes may experience performance effects from physical activity differently due to their advanced baseline cognitive and motor skillsets, emphasizing the need to tailor interventions to the unique demands of varying esports proficiency levels. This relationship suggests that while physical exercise improves general physiological and cognitive health, translating these benefits into direct esports performance requires targeted interventions addressing esports-specific cognitive and motor demands.

In this regard, especially the topic of performance parameters in esports research should be discussed further. Existing research used different approaches to determine gaming or esports performance, such as playing a certain game mode (8, 10, 17), or actual esports competition performance (18). A resulting issue is that the comparability between research results is not always given, as the methods are not yet standardized. Hence, stimuli encountered in the games or measurements used as performance measures cannot always be compared because e.g., opponents behave differently, which could influence their play and thus affect performance (62). As a result, further research should focus on whether differences or effects in performance exist in short- or long-term performance, or both.

The results of the questionnaires were significant, presenting an increase in well-being after the training bouts compared to before the training units. This finding is very much in line with literature reporting about the well-known positive effects of physical activity and exercise on well-being (63, 64). Moreover, it is of relevance as it shows the beneficial effect of physical activity on well-being for a clientele that is sedentary for several hours per day (60). In addition to the results that only three participants said they did not intend to continue with any physical activity, this study contradicts research stating that especially casual or semi-professional e'athletes are not willing to engage in physical exercise (65). Our results show that our sample supports physical activity in esports clubs, presenting a generally positive attitude toward it. This is an important finding in the context of promoting overall health and exercise in casual gaming and esports. The participants in the *STG* and *GTG* did not differ in their subjective perception of the training's effect. Both groups reported that their performance in areas such as aim, reaction, focus, rage, and stress had improved. However, it made no difference whether they trained specifically or generally. It should be noted, however, that e'athletes do not always seem to assess themselves accurately (34). Hence, these subjectively perceived improvements should be viewed with caution and were not confirmed by the objective data.

In conclusion, physical exercise improved targeted physical abilities but did not enhance short-term esports performance or suggested gaming-related parameters. However, participants reported improved well-being and a positive attitude to incorporating physical activity in esports and gaming activities. More specific training focusing on gaming-related factors like reaction or anticipation appears to have no particular advantages for gaming performance. Therefore, e'athletes are encouraged to engage in regular physical exercise, primarily to enhance overall well-being and maintain optimal health. Based on the study's findings, high-intensity interval training (HIIT) emerges as a time-efficient and effective training method that requires no equipment, making it particularly suitable for e'athletes seeking to balance fitness with their demanding schedules.

### 4.1 Limitations

The prescribed study is not free of limitations. For example, it was not possible to control whether the participants performed their training at the correct levels of intensity. However, using the CoachNow application to stay in contact with them and since both physical variables improved significantly, we are confident in assuming that participants trained with good effort. Another limitation is the fact that the sample consisted of mainly non-elite but ambitious e'athletes with on average 15.8 years of experience, who play on a regular basis. Therefore, effects of physical activity on performance might present differently in an elite e'athlete sample. However, especially with regard to the questions concerning the implementation of physical activity it can be seen as a strength as it resembles society and the kind of e'athletes who are not supported by an organization or esports club in terms of exercise and physical activity. It should also be noted that the sample size was quite small for such an intervention study, which might be the reason for the small to moderate effect sizes. Another limitation regarding the sample is that the participants were already quite physically active and engaged in different kinds of exercise on a regular basis, as well as different game genres which might have had influence on the results (61). It would be of interest to examine how results would differ in a more inactive sample, arguing that both effects of physical activity on “fitness” and gaming performance respectively would be clearer. The four-corner test used for the overall coordination assessment is a measure that might be seen as less valid than other parameters. However, this has been described earlier (45–47) and results appear to be reliable.

## Data Availability

The original contributions presented in the study are included in the article/Supplementary Material, further inquiries can be directed to the corresponding author.
