# Supplementary material for: An 8-week physical exercise intervention for e'athletes improves physical performance rather than short-term esports performance parameters - a randomized controlled trial
Source: Front Sports Act Living. 2025 Jan 21;6:1504205. doi: 10.3389/fspor.2024.1504205 (PMC11790659; doi:10.3389/fspor.2024.1504205)
Supplement: Supplementary file 1 [file Datasheet1.pdf]

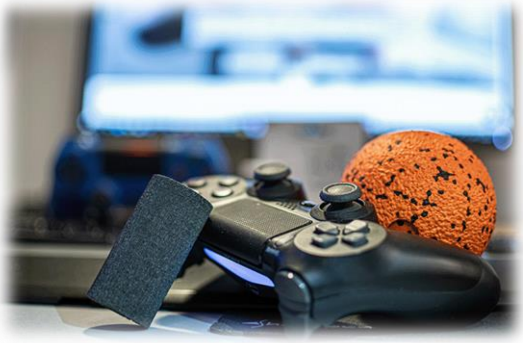

universität innsbruck

Institut für Sportwissenschaft

Training Plan Day 1

Warm-Up	2 minutes Ankle Rocking with Arm Circles [V01]<br><br>3 minutes light Jogging in place [V02]<br><br>10 light Lunges [V03]
Exercise Block 1	Tabata 1: each exercise 6 times<br>20s intensity / 20s rest = 8 minutes<br><br>A: Jumping Jack [V09]<br>B: Frontal Mountain Climber [V10]
Exercise Block 2	Tabata 2: each exercise 6 times<br>20s intensity / 20s rest = 8 minutes<br><br>A: Shadowboxing [V06]<br>B: High Knees [V05]
Cool-Down	Relaxation Circuit [V27]

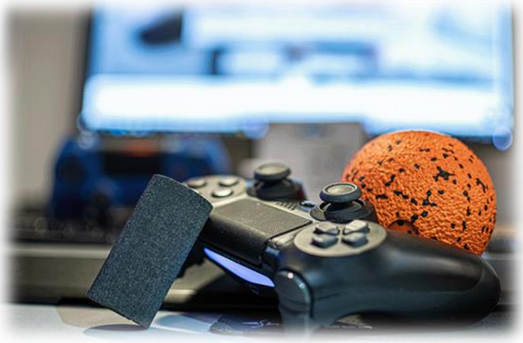

universität innsbruck

Institut für Sportwissenschaft

<u>Training Plan Day 2</u>

Warm-Up	2 minutes High Knees with Arm Circles [V12]<br><br>3 minutes light Skating in place [V13]<br><br>20 light Squats [V14]
Exercise Block 1	Tabata 1: each exercise 6 times<br>20s intensity / 20s rest = 8 minutes<br><br>A: Push-up to Stand [V04]<br>B: High Knees [V05]
Exercise Block 2	Tabata 2: each exercise 6 times<br>20s intensity / 20s rest = 8 minutes<br><br>A: Tappings [V18]<br>B: Russian Twist [V19]
Cool-Down	Relaxation Circuit [V27]

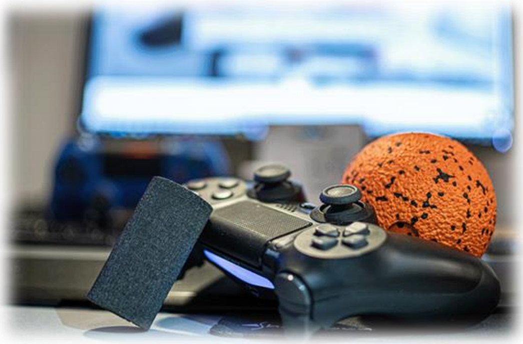

universität innsbruck
Institut für Sportwissenschaft

<u>Training Plan Day 3</u>

Warm-Up	2 minutes Side Steps with Arm Extensions (arms twice as fast as legs) [V20]<br><br>3 minutes light Jumping Rope without a rope [V21]<br><br>20 light Jumping Jacks [V09]
Exercise Block 1	Tabata 1: each exercise 6 times<br>20s intensity / 20s rest = 8 minutes<br><br>A: Squat Jumps [V23]<br>B: Side-to-Side Mountain Climber [V24]
Exercise Block 2	Tabata 2: each exercise 6 times<br>20s intensity / 20s rest = 8 minutes<br><br>A: Jump Squats [V25]<br>B: Plank [V26]
Cool-Down	Relaxation Circuit [V27]
