# Supplementary material for: An 8-week physical exercise intervention for e'athletes improves physical performance rather than short-term esports performance parameters - a randomized controlled trial
Source: Front Sports Act Living. 2025 Jan 21;6:1504205. doi: 10.3389/fspor.2024.1504205 (PMC11790659; doi:10.3389/fspor.2024.1504205)
Supplement: Supplementary file 2 [file Datasheet2.pdf]

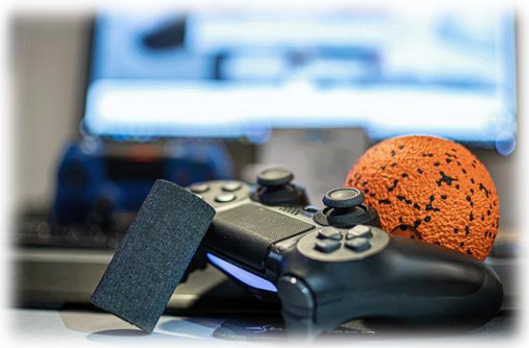

universität innsbruck
Institut für Sportwissenschaft

Training Plan Day 1

Warm-Up	2 minutes Ankle Rocking with Arm Circles [V01]<br><br>3 minutes light Jogging in place [V02]<br><br>10 light Lunges [V03]
Exercise Block 1	Tabata 1: each exercise 6 times<br>20s intensity / 20s rest = 8 minutes<br><br>A: Push-up to Stand [V04]<br>B: High Knees [V05]
Exercise Block 2	4 sets of 12 repetitions of<br>„Towel Squeeze“ [V28]<br>2 minutes rest between sets
Exercise Block 3	„Reflex Test“ with each finger, followed by alternating 5 Jumping Jacks and Squats [V07]
Exercise Block 4	30x throw a ball, close your eyes, and clap when it should hit the ground [V08]
Cool-Down	Relaxation Circuit [V27]

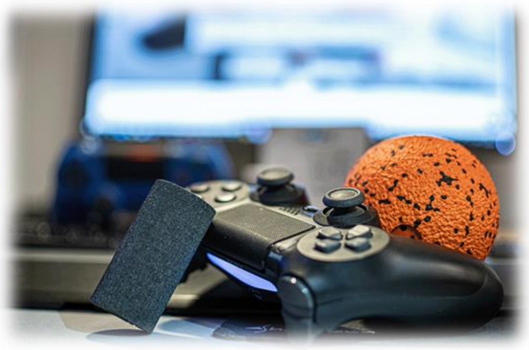

universität innsbruck
Institut für Sportwissenschaft

Training Plan Day 2

Warm-Up	2 minutes High Knees with Arm Circles [V12]<br><br>3 minutes light Skating in place [V13]<br><br>20 light Squats [V14]
Exercise Block 1	5x: 20 seconds FingerRow with Ankle Rocking and 20 seconds FingerRow with Tappings [V15]<br>30 seconds rest
Exercise Block 2	5 minutes „Color Challenge“ on your phone while alternating Lunges [V16]
Exercise Block 3	„Reflex Test“ with each finger followed by 5 Push-ups [V07]
Exercise Block 4	5 minutes left/right jumps with clapping exactly when landing [V17]
Cool-Down	Relaxation Circuit [V27]

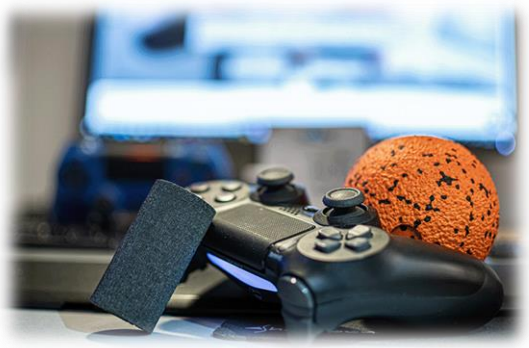

universität innsbruck
Institut für Sportwissenschaft

Training Plan Day 3

Warm-Up	2 minutes Side Steps with arm extensions (arms twice as fast as legs) [V20]<br><br>3 minutes light Jumping Rope without a rope [V21]<br><br>20 light Jumping Jacks [V09]
Exercise Block 1	4 sets of 12 repetitions<br>„Towel Squeeze“ [V28]<br>2 minutes rest between sets
Exercise Block 2	Eye video 1&2 alternately during Tabata<br>Tabata: each exercise 6 times<br>20s intensity / 20s rest = 8 minutes<br><br>A: Jump Squats [V25]<br>B: Lunges [V03]
Exercise Block 3	„Reflex Test“ with each finger, followed by 20 Skippings [V07]
Exercise Block 4	3 minutes Anticipation Video 1&2 alternately, with clapping at the midpoint during Squats [V14]
Cool-Down	Relaxation Circuit [V27]
